# A multi-pad electrode based functional electrical stimulation system for restoration of grasp

**DOI:** 10.1186/1743-0003-9-66

**Published:** 2012-09-25

**Authors:** Nebojša M Malešević, Lana Z Popović Maneski, Vojin Ilić, Nikola Jorgovanović, Goran Bijelić, Thierry Keller, Dejan B Popović

**Affiliations:** 1 Tecnalia Serbia Ltd., Vladetina 13, Belgrade, Serbia; 2 Faculty of Electrical Engineering, University of Belgrade, Bulevar kralja Aleksandra 73, Belgrade, Serbia; 3 State University of Novi Pazar, Vuka Karadžića bb, Serbia; 4 Faculty of Technical Sciences, University of Novi Sad, Trg Dositeja Obradovića 6, Novi Sad, Serbia; 5 Tecnalia, Paseo Mikeletegi 2, San Sebastian, Spain; 6 Department of Health Science and Technology, Aalborg University, Fredrik Bajers Vej 7, Aalborg, Denmark

**Keywords:** Functional electrical stimulation, Multi-pad surface electrode, Selective stimulation

## Abstract

**Background:**

Functional electrical stimulation (FES) applied via transcutaneous electrodes is a common rehabilitation technique for assisting grasp in patients with central nervous system lesions. To improve the stimulation effectiveness of conventional FES, we introduce multi-pad electrodes and a new stimulation paradigm.

**Methods:**

The new FES system comprises an electrode composed of small pads that can be activated individually. This electrode allows the targeting of motoneurons that activate synergistic muscles and produce a functional movement. The new stimulation paradigm allows asynchronous activation of motoneurons and provides controlled spatial distribution of the electrical charge that is delivered to the motoneurons. We developed an automated technique for the determination of the preferred electrode based on a cost function that considers the required movement of the fingers and the stabilization of the wrist joint. The data used within the cost function come from a sensorized garment that is easy to implement and does not require calibration. The design of the system also includes the possibility for fine-tuning and adaptation with a manually controllable interface.

**Results:**

The device was tested on three stroke patients. The results show that the multi-pad electrodes provide the desired level of selectivity and can be used for generating a functional grasp. The results also show that the procedure, when performed on a specific user, results in the preferred electrode configuration characteristics for that patient. The findings from this study are of importance for the application of transcutaneous stimulation in the clinical and home environments.

## Background

Functional Electrical Stimulation (FES) provides control signals to peripheral motor systems that are compromised after a central nervous system lesion (stroke, spinal cord injury, etc.). The use of FES combined with a splint for grasp control was introduced by Long and Masciarelli [1] to assist patients with tetraplegia. A group from the University of Ljubljana, Slovenia [2,3] suggested the use of two-channel electrical stimulation to control hand opening and closing in patients with tetraplegia. This research led to the first therapeutic application of FES, which resulted in significant carryover effects [4]. The results of these studies and many others that followed led to the development of commercial products for therapy such as the Ness H200, Bionic Glove and other similar systems [5,6].

More recently, the carryover effects of electrical stimulation have been recognized by clinicians, and several therapeutic methods have evolved [7]. Intensive exercise augmented by FES, termed functional electrical therapy (FET), provided evidence that there are rehabilitation benefits from effective electrical augmentation of movement during the period when this function is not achievable [8,9]. A therapeutic FES system comprised four pairs of relatively large surface electrodes as an interface between the electronic stimulator and tissues.

The common problems in this scenario were 1) discomfort, 2) insufficient selectivity, and 3) fast fatigue. All three of these problems could be partly eliminated by the use of multi-pad electrodes instead of large single-pad electrodes [10]. The discomfort comes from the activation of pain receptors as well as from the strong synchronous activation of all motor units within a single muscle. When large electrodes are applied, many motoneurons are activated, which results in the coactivation of various non-synergistic muscles. In short, if the electrodes are not positioned appropriately, then the desired movement will not be generated and function will not be achieved.

Finally, the firing rate of a motor unit under natural control is typically low (≈5 Hz), and a fused contraction results from the asynchronous contraction of several motor units. In contrast, FES activates many motor units simultaneously, and non-physiological frequencies (>20 Hz) are required for fused contraction, leading to a much faster occurrence of muscle fatigue.

The suggestion to use multi-pad surface electrodes was introduced in parallel with the development of multichannel electronic stimulators for arm and hand control in patients with tetraplegia [11,12]. Detailed maps for the stimulation of forearm muscles were presented by Nathan [13]. This study showed that there is major inter-subject variability.

Several research groups were involved in the design of multi-pad electrode stimulation systems along with control algorithms for various applications. The first practical system was reported by Fujii [14]. Elsaify followed this principle and suggested the use of muscle twitch response for shaping an optimal electrode [15]. A group from Eidgenössische Technische Hochschule (ETH) Zurich designed textile multi-pad electrodes with the appropriate control algorithm [16,17]. O’Dwyer incorporated wearable feedback sensors in his system and defined control based on sensory inputs [18]. The development of the "Actitrode" system [19] was part of the effort to facilitate the application of multi-pad electrodes. Detailed research led to a methodology for the minimization of non-desired movement (wrist interference when grasping) in patients with tetraplegia [20]. The results when applying the multi pad electrodes show that the required electrical charge can be delivered at current densities that do not cause more discomfort to the user than the application of large surface electrodes.

We compared the force generated via multi-pad electrodes vs. large electrodes (conventional method). The results showed that the fatigue induced by stimulation was postponed when using four cathodes instead of a single cathode over the *Quadriceps m.* of patients with chronic tetraplegia [21,22]. A charge delivered through four electrodes at low frequency asynchronously activated different motoneurons and resulted in a fused contraction. This force was similar to the force resulting from high-frequency activation via a conventional large electrode. This finding was also observed for *Triceps Surae m.* by Nguyen *et al.*[23].

**Figure 1:**
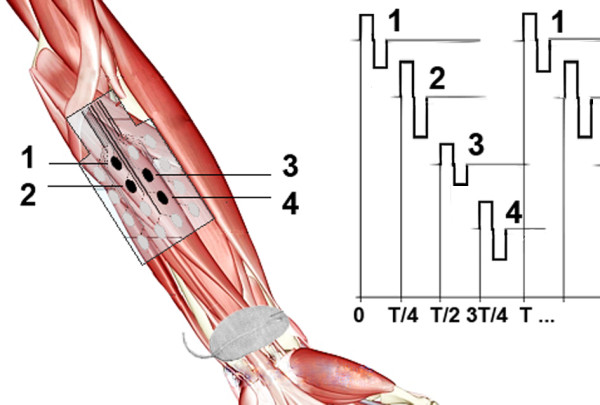
**Asynchronous stimulation of motoneurons with the multi-pad electrode.** Stimulation pulses (right panel) are time shifted and delivered to different tissues to activate portions of a single muscle or multiple muscles. The black circles (left panel) indicate selected pads that produce the desired muscle activation by sending charge to targeted regions in the tissues beneath the electrode.

We present herein a grasping control system comprising 1) a multi-pad electrode, 2) hardware capable of distributing stimulation and 3) control with feedback from wearable sensors (Figure 1). This work follows several previous studies by our group [24-27].

## Methods

### Design considerations

The application of the small pads will result in a localized electrical field that decreases spreading of currents to motoneurons of adjacent muscles or even to the same muscle. In an electrical sense, this feature enables controlled charge distribution to selected motoneurons.

If the activation of individual pads is asynchronous with short delays, then there will be a superposition of muscle forces but not action potentials. The delay between the pulses must be short enough to allow the summation of contractions. This allows the use of a lower stimulation frequency on each site and the activation of the muscle in a more physiological order, potentially postponing fatigue.

**Figure 2:**
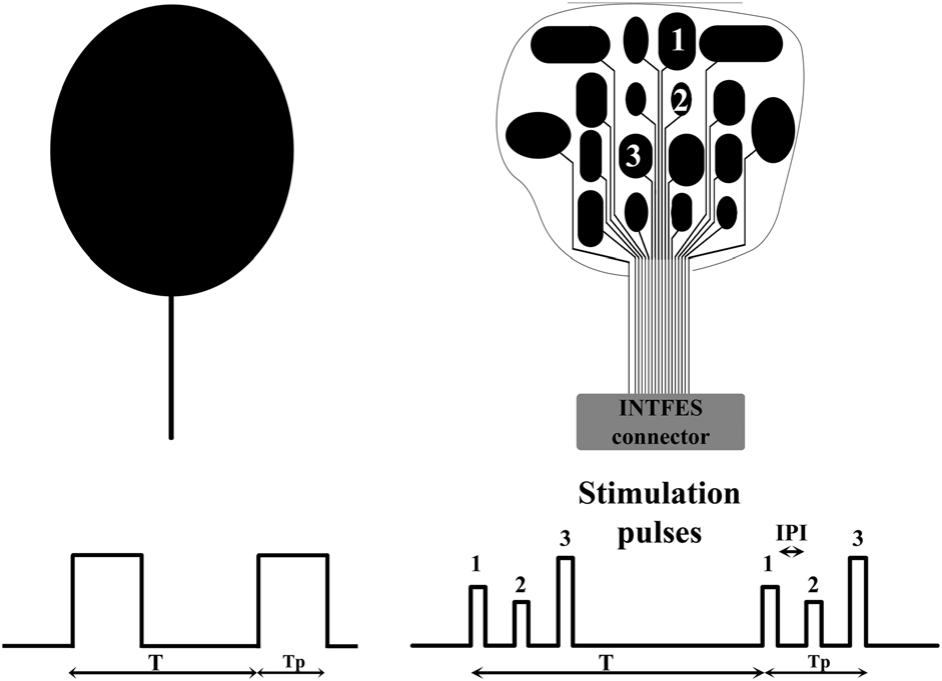
**Stimulation paradigm with single-pad electrode (left panel) and multi-pad electrode (right panel).** The multi-pad electrode has an arbitrary number and shape of pads.

We termed the new system **INT**elligent **F**unctional **E**lectrical **S**timulation (INTFES). The INTFES multiplexes charge pulses from a single pulse train to different conductive pads (Figure 2) using a fast switching system that is synchronized with stimulation pulses. This design reduces the number of high-voltage stimulator outputs to only one and allows a reduction of stimulator size, weight and power consumption.

The INTFES independently adjusts the pulses that are routed to each pad. The system controls the amplitude I; pulse duration T; Inter Pulse Interval (IPI = 1/f) within the range of 1 – T_p_/n (ms), where Tp is stimulation interval and n is the number of active pads (Figure 2); and the interval between the beginning of train pulses τ = 1/ ω (ms). The IPI and T_p_ have three distinct effects:

1. The stimulation of different motor units of a single muscle or synergistic muscles asynchronously via several pads at a high frequency f. This stimulation is achieved with the bursts of n pulses sent to n pads during a single refractory period T_p_ < 5 ms. The observed effect is the same as that of synchronous stimulation of several pads because very short T_p_ and IPI impede the occurrence of consecutive action potentials on the same motor unit. This protocol requires stimulation frequencies with ω between 20–50 Hz (τ = 50–20 ms).

2. The stimulation of different motor units of a single muscle or synergistic muscles asynchronously via several pads at lower frequency (f) with an IPI between 5 and 10 ms. In this regime, when various pads activate the same motor unit, the net effect is an “an-let” that produces a shorter muscle force-time interval and higher force compared with a single pulse stimulation of the same motor unit [28-31]. This protocol requires stimulation frequencies with ω between 20–50 Hz (τ = 50–20 ms).

3. Stimulation of different motor units of a single muscle or synergistic muscles asynchronously via several pads at low frequency ω < 20 Hz and T + IPI = 1/nω. The stimulation of each individual pad therefore contributes to the overall generated force, which can be maintained at the required level of strong fused contraction, while the frequency of action potential generation on each motor unit can be lowered to reduce muscle fatigue [21-23].

### Stimulation hardware

**Figure 3:**
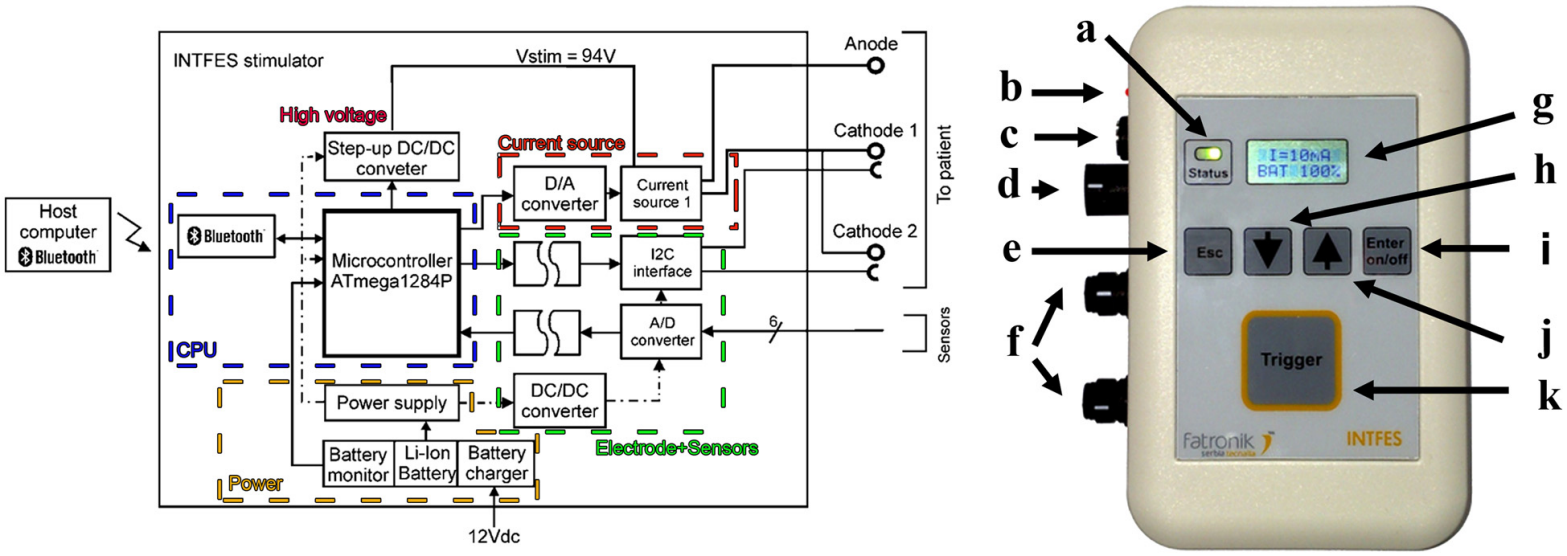
**INTFES stimulator: block diagram (left panel) and the front panel of the INTFES (right panel).** The letters at the right panel represent a) Status LED, b) Battery charger LED, c) Battery charger connector, d) Analog inputs connector, e) Esc key, f) Electrode cable connectors, g) 2-line LCD display, h, j) Arrow (↓ and ↑) keys for amplitude adjustment, i) Enter and on/off key, k) Trigger button.

*Stimulator Overview.* The architecture of INTFES (Figure 3) follows the concept of the distributed multi-pad stimulation paradigm described earlier. The stimulator output stage is a single high-voltage stage with a controlled current. The performance specifications are listed in Table 1. All of the parameters can be programmed and monitored using a program supplied with the INTFES or with a custom-made program running on a PC. Communication with the PC is realized *via* a Bluetooth interface. In addition, the stimulus amplitude can be adjusted by directly using the touch keys on the device.

**Table 1:** Stimulator technical specifications

*Dimensions*	*115 × 72 × 25 mm*
Mass	0.17 kg
Battery	7.4 V 750 mAh, integrated rechargeable Li-Ion
Operation time	> 3 hours continuous use
Battery charger input	12 V_DC,_ >500 mA, wall adapter
Battery charging time	< 3 hours
Number of channels	1
Number of multi-pad electrodes	2
Number of fields per electrodes	16
Stimulation waveform	Biphasic charge compensated, constant current pulses
Intensity (current)	0 – 50 mA (default 10 mA)
Stimulation frequency	1 - 50 pulses per second (default 50 pulses per second)
Pulse duration	50 – 1000 μs (default 250 μs)
Maximal stimulation voltage	94 V
Permitted output impedance	0 – 1.5 KΩ
Type of analog inputs	Voltage
Range of analog inputs	0 – 3.3 V
Number of analog inputs	6
ADC resolution	12 bit
Sensor power supply output	3.3 V, 100 mA
Communication type	Bluetooth 2.0 + EDR
Communication range	10 m

#### Technical specifications

*Microcontroller.* We use an 8-bit ATmega1284P (Atmel, California) operating at 11 MHz with 128 k bytes of In-System Self-Programmable Flash memory (Figure 3). This microcontroller has two wire interface (I^2^C) modules capable of a data transfer speed of up to 400 kHz, two 16-bit timers and two 8-bit timers, an SPI interface and two universal synchronous/asynchronous receiver/transmitters (USARTs). The microcontroller controls all peripherals, including the multi-pad electrodes, and it acts as a slave device to a host controller (PC, Smartphone, etc.).

*Power supply.* The INTFES device is battery powered, which protects it from hazardous current loops with other devices connected to the grid. The power supply is based on an incorporated Li-ion rechargeable 7.4 V, 750 mAh battery and includes a monitoring chip for battery capacity level. The device has two isolated parts: 1) control logic with high voltage source and 2) slave logic for controlling of multi-pad electrodes and analog inputs. This is a safety feature that eliminates the possibility of current loops between the high voltage source and an electrode printed circuit board (PCB) or malfunctioning sensor.

Charging of INTEFES occurs by an external 12 V voltage charger *via* a battery charging chip that provides optimal charging stages for prolonged battery life. To protect the user, the device cannot be turned ON during charging.

*The output stage (current control).* The output stage of the stimulator is limited to 94 V. This voltage is generated by a step-up DC/DC converter MAX773 (Maxim Integrated Products, Sunnyvale, CA, U.S.A.). The output current is generated in an H-bridge configuration that generates symmetrical biphasic pulses; its amplitude is defined using an 8-bit D/A convertor, and its duration is defined by two 16-bit timer modules in a microcontroller responsible for the direct and compensatory pulse.

*Analog inputs and multi-pad electrode control.* For safety reasons, analog inputs and multi-pad electrode control logic are isolated from high voltage circuitry with a digital circuit ADUM1201 (Analog Devices, Norwood, MA, U.S.A.) and a DC/DC power supply ISF0505A. We used MCP3208 (Microchip, Chandler, Arizona, U.S.A.) with 6 12-bit channels.

*Communications.* A Bluetooth module WT12-A-AI (Bluegiga Technologies Inc., Espoo, Finland) maintains communication between the INTFES device and the host controller. Bluetooth communication provides isolation from the grid and remote control of the device using any device with a standard Bluetooth module. Using this communication, the host can set all of the stimulation parameters, initiate a stimulation protocol, activate the pads and read analog signals and locally calculated parameters. The guaranteed delay in any read/write is 15 ms, which, for a relatively slow physiological system, permits real-time control in the INTFES stimulator or within the host controller. Thus, an algorithm can be executed by the microcontroller, but if its complexity becomes too high for the 8-bit processor, the host can co-opt some of the decision process and return command to the stimulator. In the case of recovery assessment or centralized database logging, it is possible to establish a link between the host controller application and a remote service. The Bluetooth link also allows the change of stimulator firmware.

**Figure 4:**
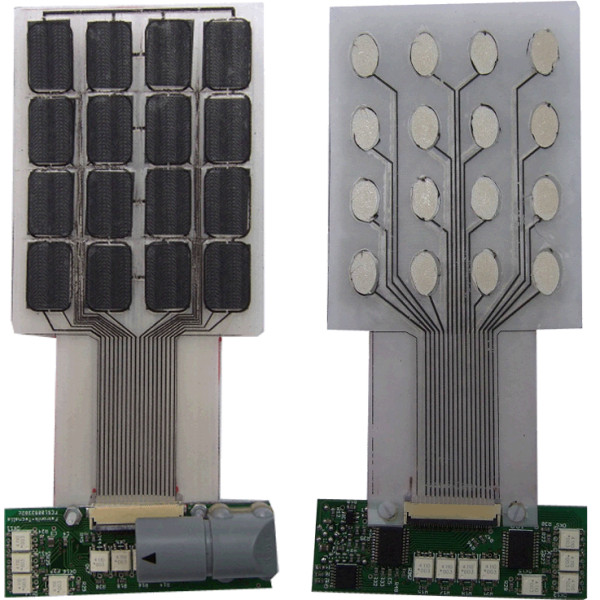
Multi-pad electrodes with the pressure sensor layer (left) and the universal contact layer covered with hydrogel (right).

*Multi-pad electrode.* The INTFES multi-pad electrode described here is custom-made for the activation of the forearm muscles responsible for grasping (Figure 4). The stimulation electrode structure comprises four layers: a polyester substrate, an Ag/AgCl electrode matrix, an insulation coating, and a conductive hydrogel. The stimulation electrode is attached to the skin with adhesive conductive gel (Axelgaard AG702 as in [32]) selected for an optimal skin-electrode interface. The relatively high impedance of the chosen hydrogel allowed us to use a single sheet of gel over all the conductive pads because high resistivity prevents lateral current spreading and steers current through the thin layer of gel. This effect was verified in healthy human experiments described elsewhere [33,34]. In parallel, the use of individual gel pads was determined to be much more comfortable than the use of a single gel pad. The custom-made electrode has an outer switching layer made of pressure-sensing material. Pressure sensors directly overlap stimulation pads and allow switch-on and switch-off of the pad at any time, as described in our patent claim [35].

To minimize the number of leads connecting the stimulator unit to the electrode, multiplexing of the stimulation pulses is performed in the electrode connector (Figure 4). The INTFES connector also contains signal conditioning electronics for the pressure sensor layer that detect pressed pads based on a defined threshold. There are seven total leads (2 for I^2^C protocol, 2 for power supply, sensory output, stimulation and enable) for any number of pads within the multi-pad electrode.

*Control software.* The control software is an application running on a host controller. The communication protocol is supported by several programming languages (C, C#, JAVA, LabView, MATLAB), enabling custom-made control software to be developed for a preferred platform. We developed user applications in a .NET environment for Windows and JAVA for Android (versions 2.3 and newer) for the setting of the stimulation parameters, and our optimization algorithm was developed in LabView (National Instruments, U.S.A.). Stimulation can be initiated or terminated promptly using control software, and it has the highest priority in the INTFES system.

**Figure 5:**
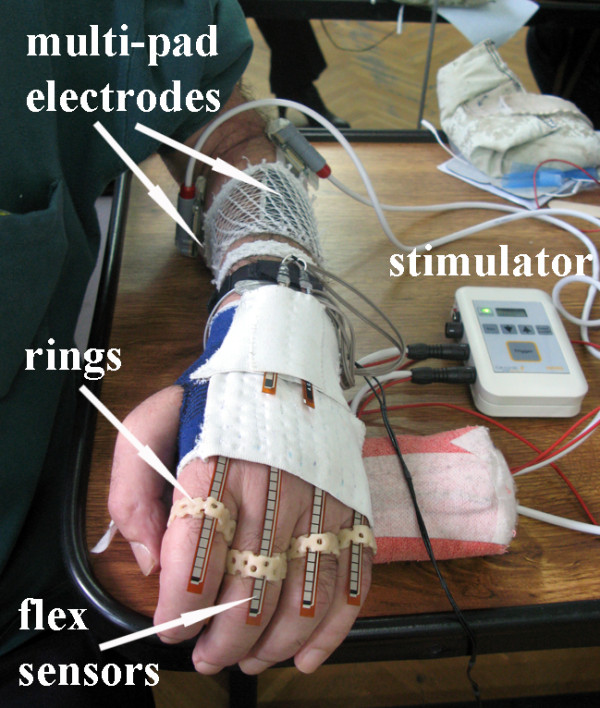
**Sensor system for the assessment of the effects of stimulation.** The flex sensors are affixed with special rings, and the glove is applied using a zipper interface. The electronics for processing are integrated into the system. The outputs are finger flexion/extension and wrist flexion/extension movements.

*Feedback sensors.* The INTFES stimulator comprises six analog inputs that can be used for online feedback control of the stimulation parameters. We designed a custom sensor system for grasp assessment. The sensors were integrated into a garment made of elastic material that allows breathing of the skin and covers only the proximal part of the hand and wrist (Figure 5) [36]. The glove had a zipper on the palmar side to facilitate mounting and positioning on the "clawed" hand (fingers flexed at the distal phalanges) in stroke patients. Because the sensors can only measure bending in a single direction, two out of six 12.5 cm long flex sensors were placed in opposition in the “pockets” sewn on the garment over the wrist to measure flexion and extension. The other four flex sensors were placed in the pockets over the four metacarpals and the metacarpo-phalangeal joints (all fingers except the thumb). To avoid creasing, we designed and fabricated special rings that have a small opening through which the flex sensors can slide [37]. The rings were fabricated of thermo plastic. One side of the rings is open to allow adaptation of the radius size according to the size of the fingers. The power supply for all of the sensors (current control) was assembled in a small box that was attached to the elastic band placed around the distal forearm. One side of the band had Velcro tape stitched on a small area on a dorsal side of the forearm. Once the garment was placed on the hand, the two sensors on the wrist were secured to this Velcro area with their own Velcro tape to prevent them from sliding out from their pockets during wrist movement.

### Stimulation software

**Figure 6:**
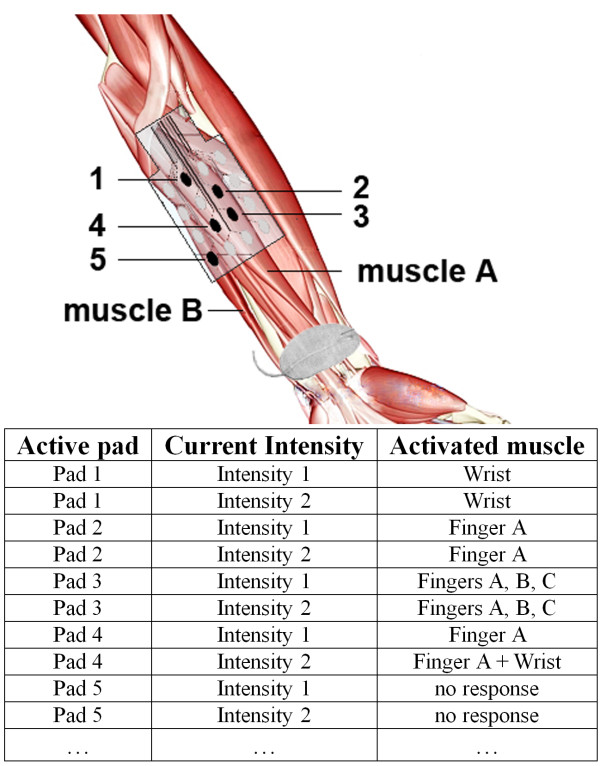
**Sample look-up table for use after the electrode active surface tuning process.** Fingers A, B and C were arbitrarily used as examples.

*Preferred electrode selection.* The first step in any application of the INTFES device is tuning the active surface (selecting the number and position of active pads and setting the current intensities in each active pad). A look-up table is provided that provides information about pad number and orientation and current intensities for desired finger movements, such as grasp generation or augmentation, with minimal wrist interference (an example is provided in Figure 6).

From the example in Figure 6, one can derive a rule-based logic that can be implemented in the grasp control algorithm. Pad 5 does not produce any response, and it should not be selected as a part of the active surface. Pads 2, 3 and 4 (Intensity 1) all move the same finger, A; therefore, these pads can be activated asynchronously with lower stimulation frequency on each pad, which can possibly postpone fatigue. Pad 1 activates only the wrist. Lower current intensities on this pad can be used to stabilize the wrist during finger extension. Pad 3 activates more than one finger, and Pad 4 produces additional activation of the wrist with higher current intensities. These pads can be included when the activation of multiple joints is required. Based on the proposed logic, after testing muscle responses for each of the electrode pads, an automated algorithm must be used to determine the preferred electrode number and position of active pads (stimulation active surface) and current intensities that produce the desired movement. The term “preferred” is dependent on the application, and in the case of grasp generation, it is used to describe finger flexion/extension and a stable wrist. “Stable wrist” is a term describing the minimal excursion of the wrist during finger opening and closing. The selection of a preferred active surface can be static, meaning one set of pads is used for each type of movement (i.e., different types of grasps), or dynamic, meaning several sets of pads are used depending on the position of the forearm. Dynamic selection (dynamic compensation) is necessary in tasks that involve movement of the skin with respect to the underlying tissue, i.e., during forearm supination/pronation. Once defined, all of the stimulation patterns can be activated by a real-time controller, reducing the number of controllable parameters in the open loop system. In closed loop systems, the derived look-up table represents the initial state of the controllable parameters, which can be further adjusted to achieve a defined goal by increasing/decreasing stimulation intensities or activating additional pads if necessary.

### Setup of a preferred electrode for patients

**Figure 7:**
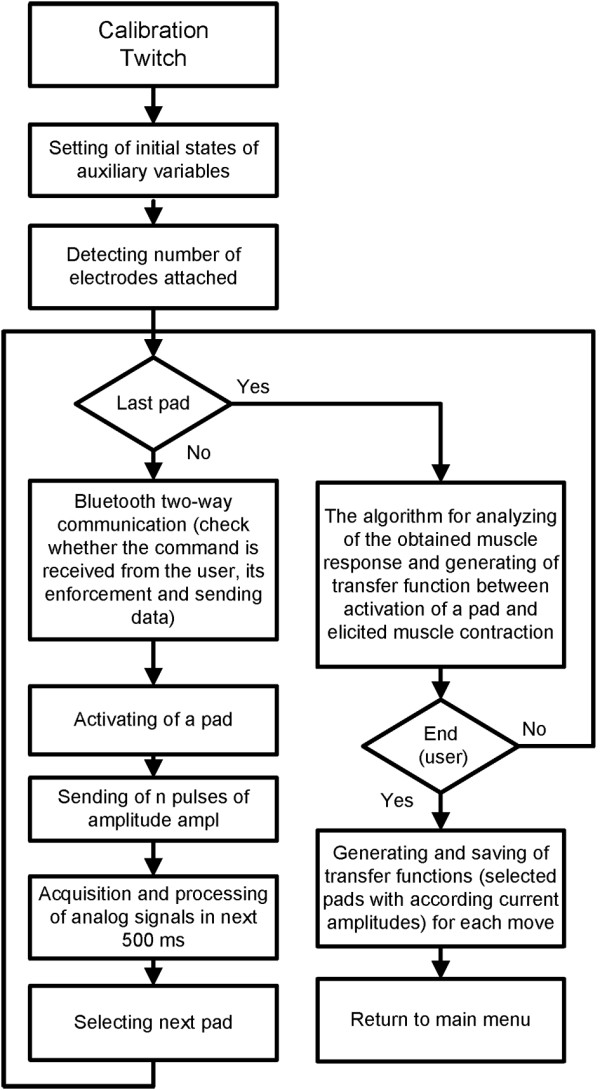
The algorithm for automatic setup.

The stimulation pattern during selection of the preferred electrode exploits muscle twitch responses generated by a short stimulation train (ST). The output was assessed by the sensor system described above. Muscle twitches that occur shortly after stimulation pulses were chosen because the output of the twitch strongly correlates with the muscle force produced by continuous stimulation [38]. The advantage of the “twitch protocol” is its short duration (<500 ms per stimulation parameter), which allows automatic testing of several stimulation intensities in a short interval. Our setup protocol is described in Figure 7 and uses 5 pulses at 40 pulses per second (pps) to each pad. The controller reads sensor outputs during the following 0.5 s. This procedure is repeated for all pads and current intensities specified at the beginning of the setup by the user. As a safety and control feature, the user can observe stimulation parameters in progress, skip pads that produce discomfort and abort stimulation. User control is implemented in the control software with visual representation of available commands.

Based on recorded sensory signals, the algorithm selects the preferred pad configuration with the appropriate current intensities. The preference algorithm is based on minimal wrist movement and large joint excursions in the fingers required for grasp. A custom subprogram in LabView 8.2 was designed to define the preferred electrode position and current intensity for effective hand closing/opening (grasp) and wrist stabilization. This routine ran automatically after completion of the stimulation protocol. To achieve this outcome, we calculated relative angular amplitudes for each stimulation train (32 pads × 5 current amplitudes = 160 stimulation trains). Based on the derived information regarding elicited movement, we employed a custom cost function for calculating grasp quality:

(1) $$\begin{array}{l} q_{f}\left(i\right)=\left(\overset{\text{small}}{\underset{\text{index}}{\Sigma }}W_{f}A_{f}\left(i\right)\overset{\text{ext}}{\underset{\text{flex}}{\Sigma }}W_{w}A_{w}\left(i\right)\right)+\\ \frac{1}{2}\left(\overset{\text{small}}{\underset{\text{index}}{\Sigma }}W_{f}A_{f}\left(i+1\right)+\overset{\text{ext}}{\underset{\text{flex}}{\Sigma }}W_{w}A_{w}\left(i+1\right)\right)+\\ \frac{1}{2}\left(\overset{\text{small}}{\underset{\text{index}}{\Sigma }}W_{f}A_{f}\left(i-1\right)+\overset{\text{ext}}{\underset{\text{flex}}{\Sigma }}W_{w}A_{w}\left(i-1\right)\right) \end{array}$$

q_f_ represents the flexion quality factor of the i-th ST, W_f_ represents the weight factor for each finger, A_f_ represents the flexion amplitude of a finger, W_w_ represents the weight factor for wrist movement, and A_w_ represents the bending amplitude of the wrist. The presented cost function takes into account neighboring ST-s if they originate from the same active pad, favoring pads to which an increase of current intensity gradually increases all flexions. If it is the first or last current amplitude of an active pad, the SC indexes become (i + 2) instead of (i-1) for the first current amplitude and (i-2) instead of (i + 1) for the last amplitude. The gradient is based on the quality factor; all pads and their current amplitudes are rated, and the pads with the largest quality factor are selected as preferred. Application of the quality factor produces a list of pads and current intensities for functional movement (as in Figure 6). A similar strategy was used for grasp optimization by Popović and Popović [20].

**Figure 8:**
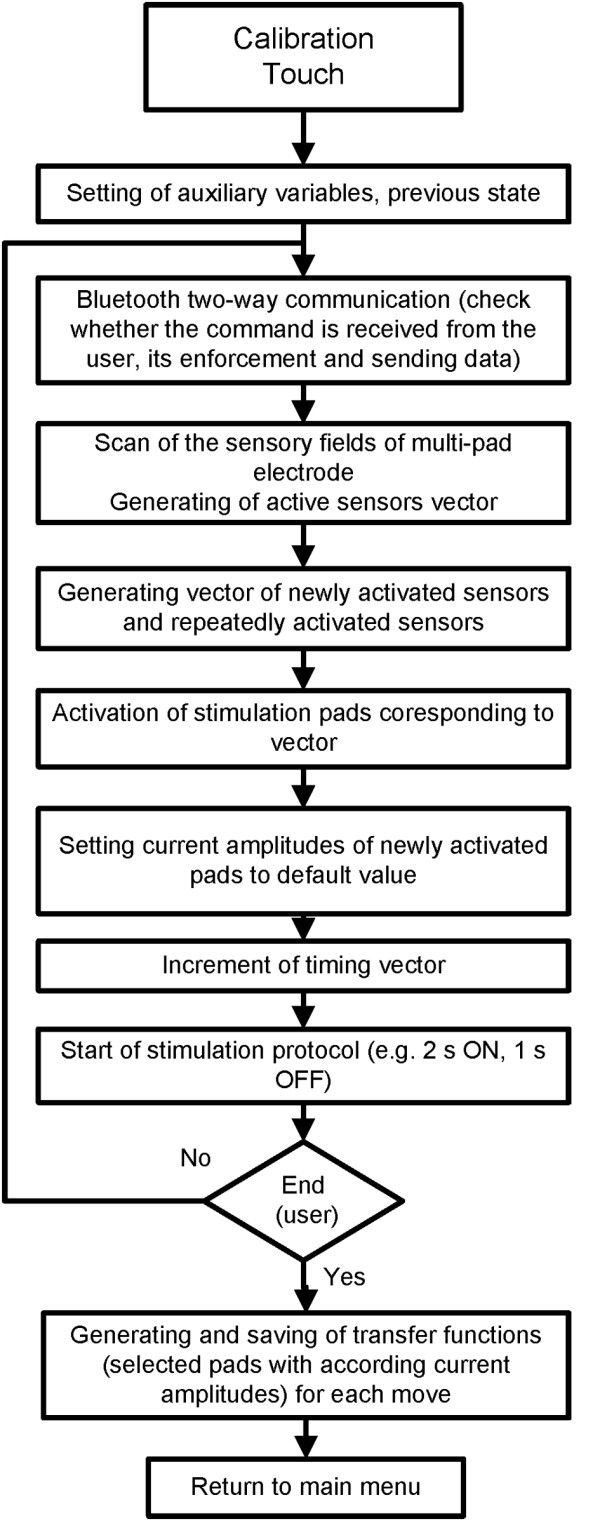
**Algorithm for the manual setup of the preferred electrode with touch sensors (adapted from **[35]**).**

As an alternative to automatic selection of the preferred electrode, the INTFES system also allows a “manual protocol” for selecting active pads and determining current amplitudes (Figure 8). This feature is designed for trained professionals and individual therapy modifications. By touching pressure sensors located on the back of the multi-pad electrode (Figure 6) as it interfaces with the patient, the clinician can activate selected pads, adjust the stimulation current and observe the response. If multiple pads are activated, stimulation pulses are distributed with a predefined IPI. A configuration of active pads producing the desired effect (e.g., hand closing) can be saved for further use. In this protocol, as in the automated one, the user has insight into the stimulation parameters while the procedure is in progress and can manually set parameters or activate/deactivate pads using a graphical user interface.

### Testing of intfes in stroke patients

The goal of this test was to estimate this system’s effectiveness and simplicity in a clinical environment and to test the effect of the preferred electrode selection procedure for hand opening and closing as part of the grasp/release procedure.

#### Subjects

Three hemiplegic patients (Table 2) volunteered for this study. They signed an informed consent form approved by the local ethics committee.

**Table 2:** Basic data for hemiplegic subjects

**Subject**	**Age (years)**	**Sex**	**Period between stroke and testing of the INTFES (months)**	**Modified Ashworth scale**	**Hemiplegia side**
**1**	48	Male	2	1+	right
**2**	51	Male	12	3	left
**3**	60	Female	31	2	right

## Results

The average time required to position the whole system, including the data glove, rings and stimulation electrodes, was less than 4 minutes [39]. The results from one patient are presented in the following figures.

**Figure 9:**
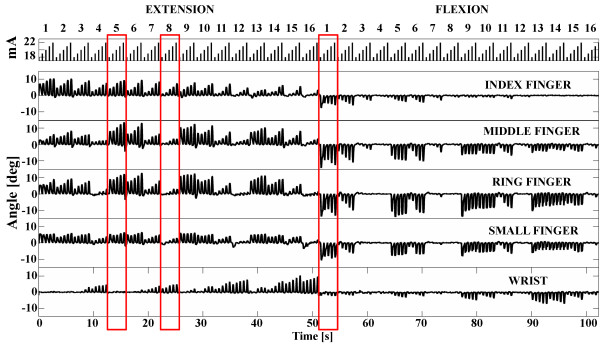
**Signals recorded with flex sensors in the twitch setup protocol.** Three marked regions are presented at a different scale in Figure 10.

**Figure 10:**
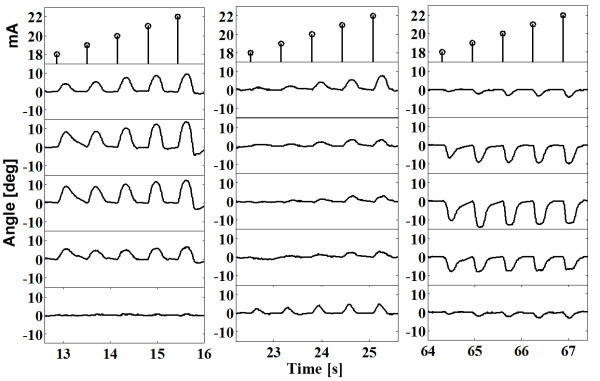
**Selected signals from Figure **9** corresponding to pads 5 and 8 on the dorsal side of the forearm and pad 5 on the volar side.**

**Figure 11:**
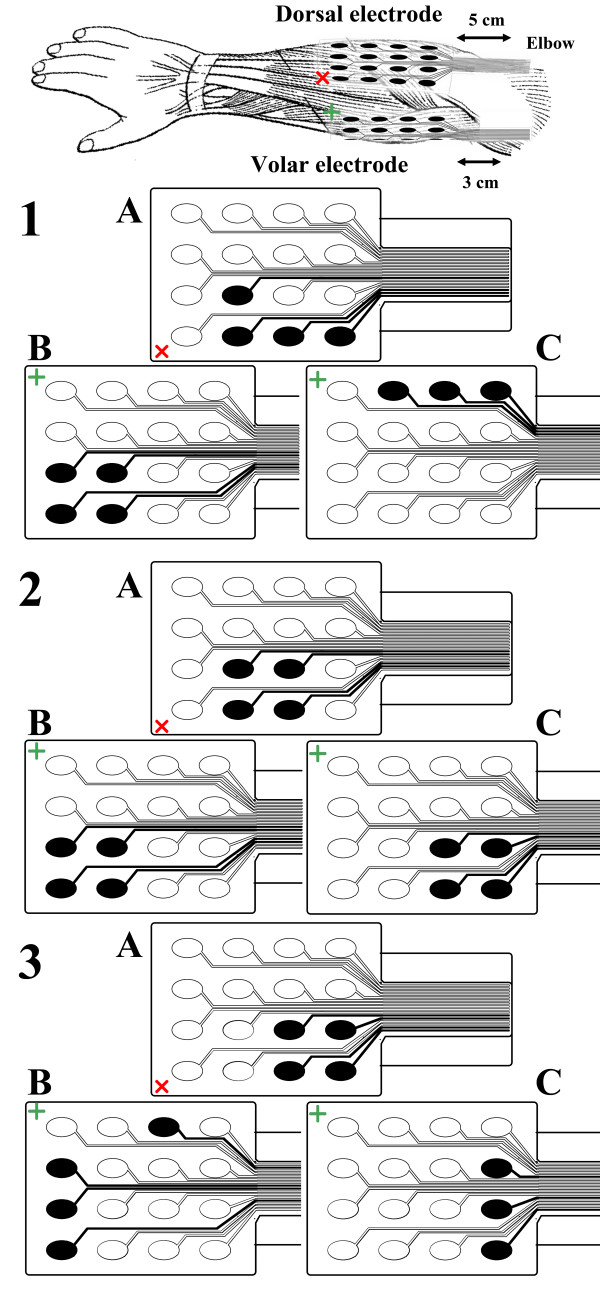
Pads selected for hand opening A (electrode placed on dorsal aspect), grasping B and wrist stabilization C (electrode placed on volar aspect) for 3 patients.

**Figure 12:**
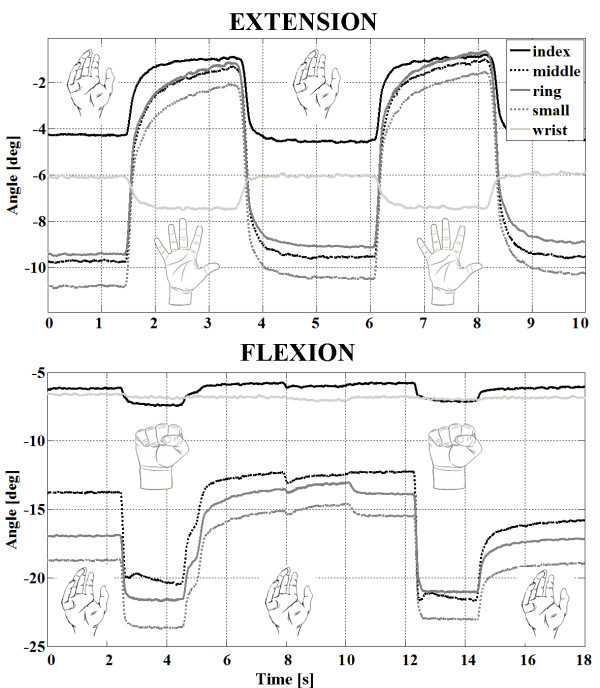
Grasp and release of patient 1 produced when the preferred pads were activated.

Figures 9 and 10 show finger and wrist twitch responses to short stimulation trains. Based on the automated pad selection algorithm described in the Procedure section (*W*_*f*_ = 1, *W*_*w*_ = −5), the optimal choice of active pads in this example would be the following: 5 (22 mA), 6 (21 mA), 9 (18 mA) and 13 (18 mA) for hand opening; 1 (22 mA), 2 (22 mA), 5 (19 mA) and 6 (21 mA) for optimal palmar grasping; and 8 (19 mA), 12 (20 mA) and 16 (21 mA) for wrist stabilization (extension) during grasping (Figure 11). To evaluate the selection algorithm, we performed hand opening and closing using the pads and current intensities indicated as preferred for these tasks. The stimulation protocol consisted of repetitive hand openings (finger extensions) and closing (finger flexions) using stimulation pulse trains at 40 Hz with a duration of 2 s. The finger and wrist trajectories during the elicited movements were assessed by the sensor glove (Figure 12).

The range of motion during evaluation testing is dependent on the patient’s neutral finger and wrist position, which varied from patient to patient. As shown in Figure 12, both the smallest flexion and extension were achieved for the index finger because the electrode size was not large enough to cover all of the hand muscle motoneurons.

The results presented in Figure 12 are for one of the patients (A). For the other two patients, we obtained similar twitch measurement results in terms of selectivity. The preferred electrode was found to be different among patients due to intersubject physiological variability (Figure 12). This finding indicates that the optimization algorithm must be applied at least one time for each patient. Repeated tests in all three patients showed that the preferred electrodes were the same if the electrode was positioned at the same position on the forearm.

## Conclusion

This pilot study in three patients clearly shows that INTFES allows wrist stabilization and selective activation of muscles that are required for hand opening and closing. Importantly, the multi-pad electrode application enables asynchronous operation [20].

The feedback coming from the sensorized garment effectively provided necessary information for the algorithm used in the tests, and the system was shown to be robust. The automated algorithm for selection of preferred grasp has been tested successfully; it used the twitch response (assessed by the flex sensors) and, in short time, automatically generated the preferred electrode configuration.

The size and shape of the electrode, in line with previous results, were found to vary substantially from patient to patient but remained the same from day to day in each patient. These results suggest that once the electrode size and shape are determined with respect to the anatomical features of the forearm, the INTFES system can be set up on the patient in less than 2 minutes by a naïve user. The results demonstrate that INTFES eliminates the tedious search process and electrode deterioration due to multiple attachments and detachments during the placement of a single pad electrode.

The preferred electrodes do not have regular shapes; they typically have a branching shape.

**Figure 13:**
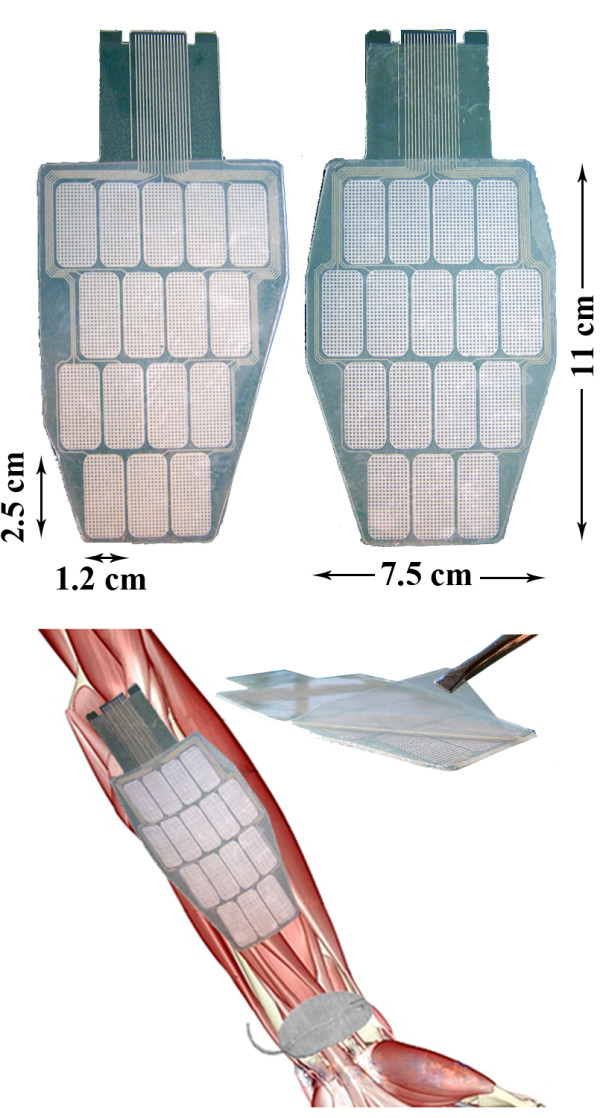
The shape of electrodes dedicated to the stimulation of wrist and finger flexor and extensor muscles.

During the testing of INTFES in these patients, some functions were difficult to achieve (e.g., index finger flexion, Figure 11). This difficulty stemmed from the inappropriate size and shape of the applied multi-pad electrode used and led to the development and design of improved electrode for the forearm (Figure 13). The electrode size and pad configuration were designed using measurements from 15 healthy subjects during hand opening and closing with minimum wrist interference.

The INTFES system is operational and is currently being used in clinical studies at the Dr. Miroslav Zotović Institute for Rehabilitation, Belgrade, Serbia. The device was well accepted by the therapists, who learned to operate the system in sessions lasting less than 30 minutes. The same system can be applied for other therapeutic modalities (e.g., foot drop correction, lower back pain, and fitness).

## Competing interests

The authors declare that they have no competing interests.

## Authors’ contributions

NM participated in design of hardware (stimulator, electrodes and sensors system), software, stimulation algorithms, clinical study recordings, data processing and drafted the manuscript. LPM participated in clinical study recordings, design of sensors system, data processing and drafting the manuscript. VI participated in hardware (stimulator) and software design. NJ participated in hardware (stimulator) design. GB participated in hardware design (stimulator and electrodes) and drafting the manuscript. TK participated in hardware (electrodes) design. DBP coordinated the study and participated in its design and drafting the manuscript.
